# Selections

**Published:** 1816-12

**Authors:** 


### PART III.

### SELECTIONS.

—— Factorum est copia nobis.

### FRENCH DICTIONARY OF MEDICAL SCIENCES.

( Continued from page 429. )

*Medical Jurisprudence.*—Article, *Avortement.* After the view already taken of this subject in its physiological and physical aspects, it now remains for us to survey it as connected with Jurisprudence. In so doing, we shall cursorily examine the light in which destruction of the fœtus has been regarded by some of the more enlightened nations, and in different ages, of the world; the degree of criminality attached to the premeditated act; and of penal severity with which its perpetration has been denounced or visited. In fact, we shall trace the judicial history of procured abortion from a remote æra to the present time; and terminate the rapid sketch by an exposition of the state of legislation respecting the crime, as it now exists among us. To the influence which sound ethics, and the adoption of a vigilant and comprehensive system of jurisprudence may exert in preventiug, or rendering less frequent, this outrage upon the best interests of society, moral and political, we shall also occasionally advert.

1. *Opinions of the Ancients respecting provoked Abortion;* and Retrospect of Legislation, as it regards this crime, to the commencement of the present Century.* The opinions and practice of some of

* By medical writers, the term Abortion is generally restricted to expulsion of the human ovum before the close of the sixth month of utero-gestation: and that of premature labour, to untimely parturition after that period. In juridical medicine, this distinction is destitute of utility. Laceration of the perinæum, we had nearly forgotten to mention as one of the signs of premature delivery. That it will not be observed in cases of abortion, or discharge of uterine hydatids, every one will be aware. We may farther remark, that clavus or the ergot of rye has been lately recommended as a remedy which may be exhibited with powerful effect to expedite the birth of the child in lingering labour, or to obviate retention of the placenta. In addition to the property which this curious vegetable substance possesses of stimulating the gravid uterus, it is said to operate with considerable certainty in restoring the suppressed menstrual discharge. *Bigelow, on the Use of Clavus, or the Ergot of Rye. New England Journal of Medicine and Surgery.* Vol. v. No. 2. See also *Journal of Science and the Arts.* No. III. EDITORS.

the more polished nations of antiquity with respect to voluntary abortion, constitute a feature, singular as dark, in the history of the human mind. The passages which, in the commencement of this article, we have quoted from the Roman poets, incontestibly prove that destruction of the human embryo was not restricted to the tent of the wandering savage, or exclusively practised upon the fruit of illicit love. By the high-born matrons of imperial Rome, in the meridian of her greatness and illumination, the most sacred claims on the female heart were remorsely violated, and the laws and interests of society, broken and sacrificed with shameless impunity. Some of the more celebrated philosophers of Greece had even recommended the employment of abortion as a mean whereby the equilibrium of population might be preserved; and the eloquent Cicero, in alluding to the case of a woman who had been condemned for destruction of her offspring, founded the justice of this decision rather upon the civil consequences, than on the moral turpitude of the crime.*

To what source shall we trace the introduction of these barbarous and unnatural prejudices among the enlightened nations of antiquity? On reverting to the civil history of these remote times, it will be found that abortion was regarded criminal or otherwise according to the doctrines of the prevailing sect of philosophy: and, by some of these, the embyro or fœtus was described as not animated by a rational and thinking soul. Now, of all the opinions of philosophers, those of the Stoics seem to have been most generally adopted by the Roman legislators: and, with an affectation of indifference peculiar to their sect, the disciples of Zeno regarded the union of soul and body as only first taking place in the act of respiration. Consequently, the fœtus, so long as it remained in utero, could not be endowed with a thinking soul. Applying, therefore, this abstruse and erroneous principle to criminal legislation, they utterly divested voluntary abortion of its atrocious character: and alike refused to the fœtus the title of a human being and the protection of human laws. It was merely named *Pars viscerum matris*, a portion of the viscera of the mother.† Thus, by metaphysical subtilties, wholly destitute of foundation in reason and experience, were the conquerors of the world governed; and thus was favoured the introduction of licentious morals, already too prevalent in the earlier ages of the Roman empire. Ideas, equally inaccurate, respecting the animation of the fœtus, probably exist among the uncivilized nations by which, at the present day, the employment of

* Memoriâ teneo quamdam mulierem, cum essem in Asia, quòd, ab hæredibus secundis accepta pecunia, partum sibi ipsa medicamentis abegisset, rei capitalis, esse damnatam: neque injuria; quæ spem parentis, memoriam nominis, subsidium generis, hæredem familiæ, *designatum reipublicæ civem*, sustulisset. *Cicero. Oratio pro A Cluent. Avito.*

† By the laws of Scotland, this obviously absurd principle is retained to the present day.

abortives is openly allowed or tolerated: or, like the women of the island of Formosa, they are impelled to the act by certain religious principles which exclude all thought or imputation of crime.

To the same pernicious direction of the human mind may be principally attributed the occasional prevalence of destruction and exposure of infants in different regions of the globe. Warlike and republican people, haunted by the groundless dread of excessive population, looked upon child-murder as a necessary evil. Aristotle even declared his opinion that, in a republic, the number of citizens should be restricted, and the education of illegitimate offspring proscribed: and by Lycurgus, this horrible precept was transformed into a law. Strabo reports, that the inhabitants of Cathea subjected their children, at the age of two months, to the inspection of a magistrate, who selected the more robust for preservation, and condemned the rest to perish. A practice, somewhat similar, was adopted by the original founders of Rome. The Celts exposed their new-born infants upon a shield to the current of a river, and considered as legitimate only those which survived this perilous ordeal. From such revolting scenes of darkness and barbarism, it is cheering to turn and contemplate a nation distinguished by its humanity. The Thebans, a people who kept prostitutes in their temples, punished with death the exposure of infants; and provided, by a special law, for the education of those children whose parents were incapable of supporting them.

By the Hebrew law, heavy penalties were denounced against the crime of procured abortion, and even death, if the mother fell a martyr to its consequences.* Constantine the Great not only directed the towns of Italy and Africa to assist those who declared themselves unable to educate their children: he instituted the most dreadful punishment against destruction of the offspring.† Under the Emperors, Severus and Antoninus, the absurd doctrine had been introduced that the fœtus only became animated at a certain period of utero-gestation. Hence, the distinction of animated and inanimate fœtus was set up: and, while infliction of an extraordinary penalty was deemed sufficient to expiate destruction of the latter, the murderer of the animated fœtus incurred the utmost vengeance of the law. But Christianity, which had operated such great changes in the legislation of Rome, exercised an equal influence on every thing relating to her morals. The successors of Constantine denounced with severity the crime of abortion; nor even admitted a distinction as to the rectitude or malignity of design with

* Exodus. Chapter XXI, verses 22—23.

† Neque gladio subjugetur, sed insutus culeo, et inter ejus furales angustias comprehensus, serpentum contuberniis misceatur, ut omni elementorum usu vivus carere incipiat, ut cœlum superstiti, terra mortuo auferatur. Cod. Theodos. C. ix. tit. 15.

which the remedies, provoking it, had been administered. Renouncing the opinions of the schools, many fathers of the church observed, that the fœtus, formless as it appears, must be animated, since it grows ; and adopted a maxim, alike salutary in its moral and political operation : *Homo est qui futurus est.* Thus the Congress, assembled in Constantinople in 692, ordained that the crime of abortion should be punished with the same severity as homicide. Yet, either from a feeling of indulgence to human frailty, or from the dominion which the doctrines of Plato long held over the fathers, the canon law was less rigorous than the Roman law, as reformed by the Emperors of the East : for by the distinction between the quick and inanimate fœtus, to which we have already alluded, the magnitude of the crime, and severity of the punishment, were judged and regulated. This distinction yet constitutes a feature in the legislation of several countries. We scarcely need observe, that it is not less erroneous in principle, than hostile in practice to sound morality, and the vital interests of the social system.

By France, as by some other nations, the reformed Roman law was adopted in all its rigour. Midwives, and in general, all medical practitioners, convicted of the crime of provoked abortion, received sentence of death. An edict of Henry II. confirmed by succeeding sovereigns, established little difference between the criminality of the female who concealed her pregnancy, or the wretch by whom a fœtus was secreted or destroyed. And letters of pardon were even sometimes requisite for the security of persons, who, ignorantly and without criminal design, had employed remedies capable of determining abortion, or been compelled to resort to this formidable expedient with a view of rescuing the mother from some more urgent peril. But the inflexible and undiscriminating spirit of the laws of Draco, which so long influenced the criminal legislation of every country where the Roman law was received, has ceased to operate. By the penal code instituted in the first years of the French republic, the crime of infanticide was made punishable by death, and that of provoked abortion, by twenty years imprisonment. " Life, in the contemplation of the *English law*, begins as soon as a child is able to stir in its mother's womb ; for, if a woman be quick with child, and any one by a potion or otherwise, killeth it in her womb, or beat her, whereby the child dieth in her body, and she is delivered of a dead child, this, though not murder, was by the ancient law, homicide ; but the modern law does not look upon this offence in quite so atrocious a light, but merely a heinous misdemeanour ; yet if the child be born alive, and afterwards die in consequence of the potion or beating, it will be murder."*

---

* See *Blackstone's Commentaries*. Vol. i. p. 129; or Male's *Epitome of Forensic Medicine*, p. 113. We wish to restrict as much as possible the limits of this historical sketch. It may, therefore, suffice cursorily to

II. *Existing Legislation of the French and English Criminal Courts, with regard to the Crime of Abortion* The French code of 1810, "more comprehensive, and better adapted to the nature of civilized man," than its predecessor, decrees, that "whoever, by food, potion, medicine, violence, or any other mean, shall have procured the abortion of a pregnant woman, either with her consent or otherwise, shall be punished with imprisonment;"—that "the same punishment shall be denounced against the woman who shall have procured abortion in herself, or consented to employ means prescribed or administered to her for such purpose, if abortion have been the consequence of them; and, lastly, that "physicians, surgeons, and other officers of health (officiers de santé), as well as apothecaries, who shall have prescribed or administered these means, shall, in the event of abortion taking place, be condemned to hard labour for a certain time."*—Finally, *the English law*, as it is at present framed, declares, that, "if any person, after the year 1803, shall wilfully and maliciously administer to, or cause to be administered to, or take any medicine, drug, or other substance or thing whatsoever, or use, or cause to be used or employed, any instrument, &c. with intent to procure the miscarriage of any woman, not being, or not being proved to be, quick with child at the time of committing such thing or using such means, then, and in every such case, the persons so offending, their counsellors, aiders, and abettors, shall be, and are declared guilty of felony, and shall be liable to be fined, imprisoned, set in and upon the pillory, publicly or privately whipped, or transported beyond the seas for any term not exceeding fourteen years." By the same Act it is ordained "that administering medicines, drugs, &c. or using means with the intent to procure abortion, *after quickening*, shall be punishable with death."†

The crime of voluntary abortion, comprehensively examined, presents some important views, upon which, although not imme-

---

observe, that, among the ancient inhabitants of Germany and Gaul, the person guilty of procuring abortion was allowed to expiate the offence by the payment of a sum of money; that, by the congress of Ancyra, in 314; of Lerida, in 524; and of Mayence, in 847; long penance, with exclusion from the Sacrament, was decreed against the crime; and that capital punishment, eternal irregularity and excommunication, were respectively denounced, by the Roman Pontiffs, in 1588 and 1591, against the principal culprit, and every priest and layman, implicated as accomplices in the charge. Sextus Quintus reserved to himself the exclusive right of absolution. By Gregory this privilege was granted to every ecclesiastic.

* See *Code Penal* (of France), § 317; *Foderé, Traité de Médecine Legale*, tom. iv. p. 386; and *Dictionnaire des Sciences Médicales*, tom. ii. *Article, Avortement.*

† See *Statutes at Large*, 43 Geo. III. cap. 28; and *Dr. Male's Epitome*, p. 114.

diately interesting to the physician in the exercise of his juridical functions, we are anxious, ere concluding this article, to direct the attention of the moral and political philosopher. Seduced by the attractions of the subject, we have already transgressed the limits originally prescribed to ourselves in discussing it; but the subject itself may, we hope, procure from the reader that indulgence and prolonged attention which our desultory and imperfect style of discussion will not be found to merit or excite.

What is the nature of the phænomenon termed quickening? and does it afford any rational ground of distinction with respect to the period of animation of the fœtus? Commonly, between the sixteenth and twentieth week from impregnation, the woman becomes sensible of a fluttering motion, sometimes visible even at a distance, in the lower part of the abdomen; and often preceded, or accompanied, by a sensation of flatulence and faintness. This is called quickening, as supposed to denote the first movement, hence to constitute the earliest sign of animation, of the fœtus. But by some physiologists different, and, in our opinion, more rational explanations of this phænomenon have been attempted.* And if, on the one hand, the axiom which we have adopted in the former part of this article be correct, that "the existence of organic life alone establishes vitality;" and if the doctrine of the venerable fathers, on the other, be undeniable in fact, as comprehensive and beneficent in operation; the distinction of inanimate and animated fœtus, introduced into the legislation of Rome, under the auspices of her emperors, and subsequently engrafted upon that of other European countries, should be utterly rejected † Sophistry, indeed, may affect to trace, with subtle finger, the boundary which separates the crime of voluntary abortion before the term of quickening, from those of destruction of the fœtus at a more mature period, and of infanticide. The unerring voice of truth and nature disclaims its acknowledgment. In fact, to the

---

* Some late writer, in one of our periodical Journals of Medicine (we forget which), has given an ingenious explanation of the phænomena of quickening. The unimpregnated uterus, it is well known, lies completely within the cavity formed by the pelvic bones. Impregnation having taken place, the organ gradually enlarges, and the time must obviously come when it can no longer be confined within such narrow limits. Arrived at a certain point of expansion, the uterus at length suddenly emerges from the pelvis into the abdomen; and thus, contends the gentleman in question, the motion, and all the train of sensations attendant on quickening, are produced.

† By some of the ancient legislators, the period of animation of the fœtus has been fixed at forty days; by some, at seven days; by others, at eighty. The fact is, vitality dates from the first stroke of the *punctum saliens*, and the future development of the fœtus is steadily progressive. Hence the rude and mis-shapen embryo has the same relations with society, the same claim upon the protection of its laws, as the more mature fœtus. Edit.

eye of reflection, child-murder, and destruction of the shapeless embryo, present a character equally atrocious; and in their influence upon the moral and physical constitution of human society, inflict a wound equally deep and malignant. Opinions, like these, cannot be too often or too strenuously inculcated on the public mind. Whether doctrines, wholly unsound in principle, can be consistently retained in practice, forms a query, which it is the province of men more deeply versed than ourselves in the science of legislation to agitate and resolve. In medicine, the solution of such a problem would require but little deliberation.

Yet, should we be disposed to deprecate any law, which, like the unsparing edict of Henry the Second of France, might regard, as equally criminal, the concealment of pregnancy and destruction of the offspring, and punish, with indiscriminate vengeance, the unhappy victim of seduction, and the monster reeking from the premeditated sacrifice of her child. A law, indeed, thus worthy of the genius of Draco, must have been founded on very superficial and inaccurate views of the female character; and will, we hope, never again disgrace an European code of legislation. Many an unfortunate woman will shrink from a humiliating confession of her shame, in the vague hope that death may intervene to conceal her wretchedness and dishonour; who would reject with disdain the proferred instrument of abortion, and rather endure the extremes of misery and neglect, than raise the weapon of destruction against the life of her offspring.

But as no dread of penal consequences can ever operate to the utter suppression of the crime, the views of a wise legislature should be directed, rather to the means of prevention than those of punishment. It would be foreign to our subject to enter formally upon this comprehensive field of discussion. We shall content ourselves with throwing out a few hints upon the means best calculated to diminish the frequency of voluntary abortion and infanticide. They will admit of separate consideration, as exercising a moral operation, or coming within the department of medical police.

Whether pregnancy be the consequence of legitimate union, or illicit intercourse, it presents an equal claim upon the interest of society. This principle, however neglected in times of ignorance and barbarism, is now acknowledged in our systems of legislation. Yet public opinion inflexibly retains its ancient severity, and continues uninfluenced by the mild and indulgent temper of modern institutions, which that principle has served to inspire. We are far from considering faulty that direction of ideas by which purity of morals, and the maintenance of social order, are promoted and secured: we wish not to see the dishonoured female and the virtuous wife placed in the same rank, and attracting equally the consideration of society. Against that rigid intolerance of the public mind we protest, which loads with exclusive infamy the victim of seduction; while the more guilty author of her wretchedness, unmolested by the execration which he merits, and little if at all degraded in public estimation, pursues elsewhere his infamous career. Some French writers have gone so far as to declare, that those countries, in which the laws of chastity are most severe, and their violation is most heavily fraught with dishonour, exhibit more frequently than others the crime of child-murder and abortion.* We shall refrain from expressing our own opinions on this subject, lest, while advocating the cause of the injured and destitute female, and urging her potent claims to mercy and commiseration, we should be suspected of a wish to sap the foundations of morality, and set up ourselves as the apologists of profligacy and crime. Nothing is more distant from our intention. How far the exertions of the legislature to facilitate and promote marriage among the inferior classes of society, by conferring peculiar privileges and exemptions on the wedded state, might operate in repressing the dreadful crime of fœticide, is a query more readily proposed than decided.

We have, secondly, to consider the influence which a vigilant system of jurisprudence may exercise in preventing the frequency of this inhuman practice. The waste of human life, resulting from voluntary abortion, or from attempts to provoke it, is, we are convinced, far greater than they whose attention has never been directed to the subject, or possess not the requisite means of calculation, will be disposed to credit. There is scarcely a town or village in the kingdom where this horrible traffic is not carried on with fearless impunity, and the uncouth instrument of some mercenary wretch occasionally employed to bring about what the midwife or emmenagogue of the druggist has previously failed to accomplish. Many young women, the victims of obscure and violent disease, annually perish from this unsuspected cause. Any thing like a remedy for this deep and insidious evil, it is in the power of Legislature alone to furnish and apply. The Act which we have last quoted, is inadequate to its suppression. Under the specious title of emmenagogues, powerful preparations of the hydrargyrus, lytta, and other violent intestinal stimulants, are yet prescribed or administered with little hazard or precaution. A law, which, rigorously enforced, should prohibit, under severe penalties, all persons from prescribing, administering, or employing any remedy, medicinal, dietetic, or mechanical, in cases of suppressed menstruation or suspected pregnancy, except by the advice or with the sanction of a regularly educated physician or surgeon, would probably go far to restrict the operation of the evil, and diminish the mortality consequent on it. That any British practitioner of medicine could so far forget the object of his art, or pollute the dignity of the professional character, as to undertake, from mercenary considerations, the work of assassination, is incredible: we will not dishonour our country by admitting the supposition.

---

* *Dictionnaire des Sciences Médicales,* tome ii. *Article, Avortement.*

An important question, relative to the subject of abortion, lastly, presents itself for our consideration. In extreme cases of malformation of the female pelvis, where delivery of the fœtus at the full term is obviously and utterly impracticable, may abortion or premature delivery be justifiably induced, and thus the life of the child be sacrificed to the preservation of the mother? This point has been long and warmly contested. The respectable and conscientious Mahon formally protests against the practice; but his arguments are ably refuted by Foderé: and most of our readers will, we imagine, concur in the opinion of Dr. Male, that it is "both legally and morally right rather to save a certain and valuable life than risk it for an uncertain one;" and that the propriety of the expedient is alike "sanctioned by humanity, policy, and justice." Nor should it be forgotten, that, in such cases, the life of the infant is not invariably sacrificed; for it sometimes, though rarely, happens, that a fœtus, born before the seventh, and even at the fifth month of utero-gestation, will live and ultimately do well. Several such cases, most respectably attested, are upon record.*

The principal monographs on abortion, in its relations with juridical medicine, are those of *Camerarius*, 4to. Tubingen, 1697; *Waldschmid*, 4to. Kilon, 1723; *Bockius*, 4to. 1726; *Schirmer*, 4to. Halle, 1729; *Alberti*, 4to. Halle, 1730; *Graff*, 4to. Halle, 1746; *Bertuch*, 4to. Halle, 1746. See also the chapters on abortion, respectively contained in the works of *Mahon*, *Foderé*, and *Male*.—Editors.

---

### FOREIGN JOURNALS.

Pathology of Abortion. *Case of provoked Abortion, terminating in Inflammation of the Uterus and Death* ‡—A delicate young woman, aged 28, of nervo-sanguineous temperament, in her second pregnancy, employed several abortive remedies without success. Disappointed in her object, she had then recourse to a

---

* A case of this kind is cited by Mahon from Brouzet. See also a case by Dr. Rodman, Edinburgh Medical and Surgical Journal, vol. xi. p. 455. We take this opportunity of calling the attention of the reader to an error which we have discovered in the commencement of this article in our last Number; and which, however obvious, we think it right to notice. In describing the *internal signs* of pregnancy and parturition, we mentioned, that they will be more strongly marked in proportion as utero-gestation is farther advanced, and will disappear shortly after delivery; but from this description the *corpora lutea of the ovaries* should be excluded, as they generally remain visible during life. Editors.

† *Dictionnaire des Sciences Médicales*, tome xv.

‡ *Observation sur un Avortement provoqué, suivi de métrite*, &c. *Bulletin de l'Athénée de Médecine de Paris*; *Fevrier*, 1816. We have thought that this history would form no inappropriate sequel to our article on Abortion. Editors.

woman, who, by means of a sharp instrument introduced into the uterus, ruptured the membranes, and detached the placenta. The operation was followed by violent colics, and by a serous and bloody discharge from the vagina.

" About fifteen hours afterwards, the patient having been partially immersed in a cold soap and water bath, was seized with general convulsions and violent pains in the hypogastrium, kidnies, thighs, and hips. In the midst of these, abortion took place about the third day. The night succeeding was restless; the uterine pains continued violent. On the morrow, October 10, 1815, pulse frequent, somewhat full, strong, not hard: agitation and pain constant and severe. *Prescribed blood-letting, an acidulous drink, hypogastric fumigations, and emollient injections.* The pains were moderated by the loss of blood. The latter was covered with a thick crust.—October 11th. Pains more severe; twitchings in the breasts, axillæ, and thighs; vomiting; pulse frequent, moderately full and strong. *Evening*: Pains considerably diminished in frequency and force. The patient much incommoded by flatus; the escape of which, *per anum*, relieves her. *Venæsection repeated.* The blood exhibited the same rich and crusty appearance as before.—October 12th. Passed a tranquil night. Pressure on the hypogastrium not painful; tumefaction and pain of the mammæ; lochial discharge continued; pulse very frequent and little developed. *Evening.* Fever slightly marked; tongue very red; hypogastrium rarely affected with the pains, which now seem to be concentrated about the breasts.—October 13th. Thirty-six liquid and yellowish stools passed during the night, with umbilical pains, but unattended by griping or tenesmus; pains in the pubic region and mammæ, the tension of which is increased; discharge from the vagina, of a white and slightly bloody fluid; countenance neither dejected nor flushed; tongue very red and moist; pulse frequent and feeble. *Prescribed an emollient decoction, and broths, internally; and a poultice to the hypogastrium. Evening.* Twelve more stools; abdomen swollen; borborygmi; pains about the navel; twitchings in the mammæ; hypogastrium painless on pressure; pulse more frequent, small, feeble. Nine or ten stools during the night; sleepless.—October 14th. Stools less frequent; no abdominal pains; breasts subsiding; tongue constantly red; lochial discharge more abundant. One stool only during the day; face flushed; thirst; considerable heat; fever more conspicuous towards the evening.—October 15th. Diarrhœa returned; mammæ completely shrunk; no ventral pain; lochia continued; an erysipelatous eruption on the face, chest, arms, and lower limbs; thirst; nausea; pulse very frequent and contracted. *Evening.* Wandering pains of the epigastrium and hypochondria, kidnies, and groins; vomiting of a greenish matter; face pale; pulse small and feeble; profound dejection.—October 16th. Little sleep; fluid, resembling solution of acetate of copper, several times vomited; no abdominal pains; transient appearance of nettle-rash on the arms and lower limbs; pulse less frequent, not hard. Four stools duringthe day ; tongue less red ; lochia continuing. *Emollients, broths, internally ; cataplasm to the hypogastrium, and opiate epithem to the epigastric region.*—October 17th. During the night disturbed sleep : several stools. *Morning.* Confused ideas ; tongue dry in the middle, moist on the edges, less red generally ; stupor commencing ; abdomen swollen ; no pain ; lochia continue; pulse frequent, readily compressible. *Evening.* Erysipelatous rash universally ; slight coma ; left eye completely closed ; lochial discharge suppressed ; abdominal tension aggravated. *Frictions with extract of cinchona and camphor, with volatile liniment. Stimulant pediluvia and sinapisms to the feet ; chamomile fomentations to the abdomen. Blister to the internal part of each thigh.*—October 18th. Violent delirium during the night, and subsultus tendinum. *Morning.* Alternation of drowsiness and slight delirium ; eye-lids completely closed; countenance altered ; tongue red, dry ; loss of consciousness. Pulse frequent, weak, and regular. Blistered surfaces discoloured. *Evening.* Debility increasing. Slight serous and bloody discharge from the vagina. *Sinapisms to the thighs ; eight leeches to the interior of the labia pudendi ; poultice to the hypogastrium ; chamomile injections : wine, cordials, lemonade, internally.* October 19th. Borborygmi, and copious stool, after an injection ; abdomen reduced to its natural size. Constant delirium ; anxiety; decomposition of the features; eyes half open and turned up ; tongue and lips parched and sooty ; severe pain in the hypogastric and iliac regions. No vaginal discharge. *Dry cupping on the internal part of the thigh ; six leeches to the hypogastric and iliac regions ; emollient fomentations ; lemonade ; wine ; sinapisms to the feet.*—October 20th. Profound coma; features more decomposed ; rigidity of the superior limbs and cervical muscles ; deglutition difficult; respiration somewhat abdominal ;* several stools. The patient was plunged *into a tepid bath ;* but, in about five minutes, the countenance becoming discoloured, and the pulse sinking, she was replaced in a warm bed. At seven o'clock, P. M. the face grew deathly ; the corneæ dim and wrinkled ; the nose sharp. The senses were completely lost ; rigidity of the arms somewhat decreased; deglutition constantly difficult ; respiration more frequent and laborious ; pulse rapid and thready, not irregular. Death took place at four o'clock on the next morning."

" *Dissection twelve hours after death.* Abdomen considerably distended, and yet warm ; putrefaction of the external genital organs commencing ; the whole vulva of a blackish slate colour, and exhaling an offensive smell. The interior of the vagina presented the same appearance, but less strongly marked. On opening the abdomen, about one pint of purulent fœtid serum was found in

* Executed rather by the abdominal, than by the intercostal, muscles. EDIT.

the pelvis. The sheet of peritonæum lining this region was red, thickened, minutely injected, and covered, here and there, with a puriform albuminous exudation. The traces of inflammation of this membrane were no where so conspicuous as on the uterus and its appendages. This organ was nearly as large as the fist; and its parietes scarcely less than an inch in thickness. Its fleshy substance was not more red or dense than natural, and displayed no vestige of inflammation. The body and cervix formed but one mass, the orifice of which was sufficiently dilated to admit the extremity of the finger. Neither wound nor cicatrix was perceptible; but merely some inequalities, and a large violet-brown spot extending to the interior, and in no wise resembling an eschar. The left ovary, of the volume of a large nut, was filled with pus and readily lacerable. It was intimately adherent to the fimbriated extremity of the Fallopian tube, and to the omentum; which, in this place only, shared the inflammation. The right ovary also had suppurated, but was less disorganized than the other. Several of the intestinal convolutions were, at the point of contact with the uterus and its appendages, red, thickened, and covered with pus. Elsewhere, the intestinal tube was sound; its mucous membrane universally white as linen, and exempt from morbid alteration. The stomach, liver, and spleen were healthy. The thorax and cranium were not inspected."

---

ANIMAL CHEMISTRY. — *On the colouring Principle of the Blood of Animals.**

" I. *Historical Sketch of the opinions entertained by physicians and chemists previously to Mr. Brande, on the nature of the coulouring principle of the blood.*—Lemery first demonstrated, by experiment, the presence of iron in the blood. Menghini subsequently endeavoured to determine the relations of this metal with the animal fluid."

" From this period, physicians and chemists have generally attributed the colour of the blood to iron; but as this metal is, by itself, insoluble in the animal fluids, chemists have sought in the blood for a substance capable of effecting this office; and some have believed that they have discovered it in the soda which exists in small quantity in the blood†: others, from the fact of the blood yielding, upon incineration, sub-phosphate of iron, have referred this function to the phosphoric acid.‡

" But such opinions being liable to objections which their authors themselves have not dissembled, this interesttng point of doctrine has been submitted to a fresh examination; and Mr. Brande

---

* *Note sur le principe colorant du Sang des Animaux.* Par M. Vauquelin. *Annales de Chimie et de Physique, Jan.* 1816.

† Deyeux et Parmentier.

‡ Sage, Gren, Fourcroy, et Vauquelin.

has had the honour of first demonstrating, by direct experiment, that the cause of the colour of the blood resides in a peculiar animal matter, and not in iron, as was before imagined.*

"I have thought that a discovery so interesting alike to chemistry and physiology deserved confirmation by experiment; and I hope that, even if nothing new be added to the facts announced by Mr. Brande, I may at least claim the merit of having recalled to it the attention of the learned world. I, at first, repeated most of the experiments of the English chemist, and found them exact. I have made others which are new; and particularly sought for a simple method of obtaining the colouring principle of the blood in a state of purity."

"II. *Process for obtaining in a state of purity the colouring principle of the blood.*—Take the coagulum of blood well drained upon a hair sieve; bruise it in an earthen vessel with four parts of sulphuric acid diluted with eight parts of water; and heat the mixture, for five or six hours, to the temperature of 70° Centig. (about 157° Fah.) Filter the liquor while yet warm, and wash the residue with warm water equal in quantity to the acid employed: evaporate both till they are reduced to one half; then pour in ammonia until there remains only a slight excess of acid. After having shaken the liquor, let it rest awhile, and a sediment of a red purple colour will be obtained.

"Decant the liquid as soon as it is clear, and repeatedly pour water on the residue, until the last portion no longer precipitates nitrate of barytes.

"The precipitate, thus washed, should be put upon a filter; and when it is completely drained on bibulous paper,† let it be removed with an ivory knife, and put into a capsule to dry. This is the pure colouring part of the blood. The process, just described, seems to me more simple and certain, than those proposed by Brande and Berzelius.

"III. *Reflections on what takes place in the preceding operations.* — The sulphuric acid, in dissolving the colouring matter of the blood, dissolves at the same time, a considerable quantity of albumen, and probably of fibrine; but these substances remain in solution in the liquid after the colouring principle has been precipitated by ammonia.

"The coagulum of blood, treated thrice by the same quantity of sulphuric acid as was first employed, appears yet as much coloured as ever, and the acid, boiled with it, is almost as deep-coloured; which proves that a great quantity of acid is necessary to dissolve the colouring matter of the blood; at least, if there be not two sorts of colouring matter differently soluble.

"It seems that albumen opposes the solution of the colour of

---

* *Annales de Chimie*, Tom. xciv.

† The original is *papier joseph*, a term which we do not precisely comprehend. Ed.

the blood in acids; for this latter, when pure, dissolves very abundantly in them.

"IV. *Properties of the colouring principle of the blood.*—It has neither perceptible smell nor taste. Mixed with water, it has a red vinous colour; but does not dissolve in it. When dried, it appears black as jet, of which it presents both the fracture and lustre. Thus dried, it dissolves readily in acids and alkalies, and communicates a red purple colour to these solutions. Its solution in muriatic acid does not render turbid the solution of muriate of barytes; which proves, that when well washed, it retains no sulphuric acid. Pure gallic acid, and prussiate of potash, effect no change in the colour of the acid solutions of this substance; *which proves that it contains no iron;* while in the liquid, from which this substance has been precipitated, the two re-agents in question instantaneously shew the presence of iron in considerable quantity. The infusion of nut gall, which contains tannin, precipitates the solution of the colouring matter in an acid; but does not change its colour. Exposed to the action of fire in a close apparatus, it suffers no change of form or colour; it exhales a smell resembling that of animal substances, furnishes carbonate of ammonia and a red purple oil, but scarcely any gas. After the exposure to heat, it no longer dissolves in acids or alkalis; but is reduced to a carbonaceous state. As the volume of this substance does not perceptibly alter during the process, it must contain much carbon. This substance being insoluble in water, the blood must necessarily contain something capable of effecting its solution; and probably this is alkali, a very small quantity of which will suffice to dissolve this substance. Yet, as the colour of the blood is deposited, in time, from the fluid in which its coagulum is washed, it should seem to be merely suspended. The solution of the colouring principle of the blood in nitric acid diluted with water, suffers no change of colour: nitrate of silver does not disturb it; but acetate of lead forms a brown precipitate, and completely removes the colour. The coagulum of blood which has been, several times, boiled with the sulphuric acid, dissolves completely in a small quantity of potash, from which muriatic acid entirely precipitates it, at least if there be an excess of the acid: the solution then preserves a red colour. When by repeated washing in cold water, the greater part of sulphuric acid has been separated from the mass of blood, the residue dissolves abundantly in warm water; but the solution which results from it, has a brown, instead of a red colour. The albumen of the blood, which contains the colouring matter, is deposited on resting for a certain time, and the liquid becomes greenish yellow. But if this colouring matter remain in the albumen until decomposition is commencing, it then re-dissolves, and the liquid resumes a scarlet colour; for the ammonia, which is developed during putrefaction, produces this effect, and the solution, which is red, becomes scarlet on mixing with the yellowish albumen. If two parts of cold alcohol are poured on the albumen of beef, and, after the liquor has been filtered, and the coagulum drained, it is boiled with seven or eight parts of fresh alcohol, the latter acquires a fine citron yellow colour. Lastly, if the same operation be thrice or four times repeated, the alcohol no longer is coloured, and the albumen becomes white. The alcohol, evaporated in a retort, leaves a fat oil of yellow colour, of mild taste, and of soft consistence. From the experiments of Mr. Brande, and my own, which afford them a confirmation, of which, in truth they have no need, the colour of the blood is owing to a peculiar animal substance, produced by the vital powers, and particularly by the influence of respiration; and the opinion of physicians and chemists, who, up to the present period, have attributed this property to the presence of iron, must be abandoned; at least, as to its being the sole cause; since this substance may be obtained in a separate state, exempt from the metal."

"*Reflections.*— Although we extract from blood, by the means just announced, a colour in which the most delicate experiments discover not the slightest trace of iron; yet it must be acknowledged. that the colour of this substance differs much from that of the blood itself. The latter, as every one knows, is of a bright red hue, analogous to scarlet. The substance in question, when separated from the blood, has a red purple, and even violet colour, which appears greenish by refraction."

It is true, that the blood, deprived for some time of the influence of the air, contracts a purple vinous colour, considerably resembling that of the separated colouring principle; but as soon as this blood is again exposed to the air, it resumes the pristine vermilion hue. The colouring principle, on the contrary, remains unaltered by the air.

Does this substance suffer any alteration from the acids and heat which we are compelled to employ in separating it from the other substances which enter into the composition of the blood? Or to the mixture or combination of this principle with the other elements of the blood is the colour of the latter attributable?

"If the existence of the oil which we have before mentioned, be constant in the blood of man and (other) animals, this fluid must be composed of four essential and constituent elements; to wit: albumen; fibrine; colouring matter; a fat and soft oil."

I have, in imitation of Mr. Brande, attempted to fix upon cotton, by means of different mordants, the colouring matter of the blood, dissolved either in acids or alkalis; but I have obtained from it nothing fine or solid. I doubt whether this substance can ever be successfully employed as a dye.

