# Errata

**Published:** 1816-07

**Authors:** 


### ERRATA.

P. 504, second Note. *Read*, carbonate of copper, copper partly metallic, &c.

